# Characterization of the antibody response to SARS‐CoV‐2 in a mildly affected pediatric population

**DOI:** 10.1111/pai.13737

**Published:** 2022-02-21

**Authors:** Sonja Kopanja, Pia Gattinger, Klara Schmidthaler, Justyna Sieber, Katarzyna Niepodziana, Thomas Schlederer, Lukas Weseslindtner, Karin Stiasny, Florian Götzinger, Winfried F. Pickl, Thomas Frischer, Rudolf Valenta, Zsolt Szépfalusi

**Affiliations:** ^1^ Division of Pediatric Pulmonology, Allergy and Endocrinology Department of Pediatrics and Adolescent Medicine Comprehensive Centre of Pediatrics Medical University of Vienna Vienna Austria; ^2^ Department of Pathophysiology and Allergy Research Division of Immunopathology Centre for Pathophysiology, Infectiology and Immunology Medical University of Vienna Vienna Austria; ^3^ Department of Clinical Immunology Wroclaw Medical University Wroclaw Poland; ^4^ Centre for Virology Medical University of Vienna Vienna Austria; ^5^ Department of Pediatrics and Adolescent Medicine Klinik Ottakring Vienna Austria; ^6^ Institute of Immunology Center for Pathophysiology, Infectiology and Immunology Medical University of Vienna Vienna Austria; ^7^ Karl Landsteiner University of Health Sciences Krems Austria; ^8^ Sigmund Freud Private University Vienna Austria; ^9^ Laboratory for Immunopathology Department of Clinical Immunology and Allergology Sechenov First Moscow State Medical University Moscow Russia; ^10^ NRC Institute of Immunology FMBA of Russia Moscow Russia

**Keywords:** antibodies, children, COVID‐19, humoral immunity, pediatric population, SARS‐CoV‐2, virus neutralization

## Abstract

**Background:**

While children usually experience a mild course of COVID‐19, and a severe disease is more common in adults, the features, specificities, and functionality of the SARS‐CoV‐2‐specific antibody response in the pediatric population are of interest.

**Methods:**

We performed a detailed analysis of IgG antibodies specific for SARS‐CoV‐2‐derived antigens S and RBD by ELISA in 26 SARS‐CoV‐2 seropositive schoolchildren with mild or asymptomatic disease course, and in an equally sized, age‐ and gender‐matched control group. Furthermore, a detailed mapping of IgG reactivity to a panel of microarrayed SARS‐CoV‐2 proteins and S‐derived peptides was performed by microarray technology. The capacity of the antibody response to block RBD‐ACE2 binding and virus neutralization were assessed. Results were compared with those obtained in an adult COVID‐19 convalescent population.

**Results:**

After mild COVID‐19, anti‐S and RBD‐specific IgG antibodies were developed by 100% and 84.6% of pediatric subjects, respectively. No difference was observed in regards to symptoms and gender. Mounted antibodies recognized conformational epitopes of the spike protein and were capable to neutralize the virus up to a titer of ≥80 and to inhibit the ACE2‐RBD interaction by up to 65%. SARS‐CoV‐2‐specific IgG responses in children were comparable to mildly affected adult patients.

**Conclusion:**

SARS‐CoV‐2 asymptomatic and mildly affected pediatric patients develop a SARS‐CoV‐2‐specific antibody response, which is comparable regarding antigen, epitope recognition, and the ability to inhibit the RBD‐ACE2 interaction to that observed in adult patients after mild COVID‐19.

**Abbreviations**
Ab: antibody
ACE2: angiotensin‐converting enzyme 2
ARDS: acute respiratory distress syndrome
COVID‐19: coronavirus disease 2019
ELISA: enzyme‐linked immunosorbent assay
HRP: horseradish peroxidase
IgG: immunoglobulin G
ISU: ISAC standardized unit
MIS‐C: multisystem inflammatory syndrome
NP: nucleocapsid protein
OD: optical density
RBD: receptor‐binding domain
S: spike protein
S1: spike protein receptor‐binding subunit
S2: spike protein membrane fusion subunit
SARS‐CoV‐2: severe acute respiratory syndrome coronavirus type 2
VNT: virus neutralization test

**Key Message**

Age‐dependent diversity in course of COVID‐19 was repeatedly observed, with children presenting rather mild or no symptoms and adults having more severe manifestations. Here, we performed an in‐depth analysis of antibody responses in 26 previously reported seropositive children and an age‐ and gender‐matched control group. After a mild course of COVID‐19, children developed anti‐S and anti‐RBD‐specific IgG antibodies directed towards conformational epitopes on spike protein that were neutralizing the virus and inhibiting ACE2‐RBD interaction. The specificity and magnitude of the antibody response were comparable between children and adults after mild COVID‐19, implying that the age‐related clinical discrepancies might not be related to divergent properties of antibody response, as previously thought.

## 1 INTRODUCTION

Rapid spreading and high mortality rates made coronavirus disease 2019 (COVID‐19) the most severe respiratory pandemic since the Spanish flu. Although need for the development of therapeutic strategies urged rapid growth of knowledge about the severe acute respiratory syndrome coronavirus 2 (SARS‐CoV‐2) immune response, data for the pediatric population remained scarce.

The clinical manifestation of COVID‐19 ranges from asymptomatic and mild course to severe respiratory impairments that may lead to death. Interestingly, an age‐related difference in disease presentation was observed.^1^, ^2^, ^3^ The older population was more prone to develop severe outcomes such as an acute respiratory distress syndrome (ARDS),^4^, ^5^, ^6^ while the majority of children and young adults had mild to moderate clinical manifestations and rarely developed a severe course of disease in a form of the multisystem inflammatory syndrome (MIS‐C) or thrombotic complications.^1^, ^7^, ^8^, ^9^ This age‐related discordance in the disease course is one of the most striking findings and may have important implications for the development of preventive and therapeutic strategies against SARS‐CoV‐2 infections. Several hypotheses explaining this phenomenon were suggested. One possibility may be different immune responses to SARS‐CoV‐2 in children and adults. It has been described that children had a reduced breadth of anti‐SARS‐CoV‐2 antibodies, predominantly mounting anti‐S but not anti‐NCP specific IgG, and had reduced virus‐neutralizing activity.^2^ Furthermore, predominant activation of innate immune response represented in a pediatric population was associated with a diminished release of proinflammatory cytokines and a more favorable clinical course of the disease.^10^, ^11^ Another mutually nonexclusive possibility for the mild course of COVID‐19 in children could be a lower expression of the angiotensin‐converting enzyme 2 (ACE2) receptor, which is recognized by the receptor‐binding domain (RBD) of SARS‐CoV‐2.^12^, ^13^, ^14^, ^15^ Furthermore, it has been suggested that ACE2 expression could be affected by co‐morbidities, which may be associated with severe courses of COVID‐19.^16^

In majority of the SARS‐CoV‐2 convalescent adult patients, antibodies to S, RBD, and NCP were detected.^2^, ^17^, ^18^, ^19^, ^20^ Further investigation revealed that neutralizing antibodies recognize conformational, but neither linear epitopes nor S‐derived peptides.^20^ Interestingly, several groups reported additional non‐RBD epitopes on the S1 that could be involved in virus neutralization.^21^, ^22^, ^23^ It is still debated whether children develop antibodies to the same extent and recognizing the same epitopes as adults.

Here, we performed an in‐depth analysis of the IgG antibody responses to S and RBD SARS‐CoV‐2‐specific antigens and S‐derived peptides by ELISA and microarray technology in representative samples from a previously undertaken study in a cohort of more than 2000 schoolchildren.^19^ Additionally, we investigated the virus‐neutralizing activity of antibodies and their ability to inhibit the interaction of RBD and ACE2 in a recently developed molecular interaction assay.^24^ Moreover, we compared results obtained in children with data from a SARS‐CoV‐2‐affected adult population collected at the same time period.^20^, ^25^

## 2 METHODS

### 2.1 Study population

In a recent cross‐sectional study, we performed a comprehensive seroprevalence analysis in a population of 2069 schoolchildren in Vienna, Austria.^19^ The study was conducted from May to July 2020, while the wild‐type variant of SARS‐CoV‐2 was the most dominant. In this study, children were classified as SARS‐CoV‐2 seropositive when they first tested positive in the Wantai SARS‐CoV‐2 total Ab ELISA (Beijing Wantai Biological Pharmacy Enterprise, China), and an in‐house neutralization assay or the combination of the SARS‐CoV‐2‐NCP‐IgG ELISA and the Anti‐SARS‐CoV‐2 ELISA (both Euroimmun, Lübeck, Germany) concordantly confirmed the presence of SARS‐CoV‐2‐specific antibodies.^19^, ^26^ Children with negative Wantai SARS‐CoV‐2 total Ab ELISA test results were not further tested and defined as seronegative.

In the present study, we performed an in‐depth characterization of SARS‐CoV‐2‐specific antibody responses in 26 seropositive and 26 age‐ and gender‐matched seronegative subjects (controls) from the previous study. The study was approved by the Institutional Review Board of the Medical University of Vienna (EK No.: 1401/2020).

Pre‐pandemic sera for historic controls (HCs) were provided from the serum bank of the Division of Immunopathology, obtained between 1996 and 2019 were used for establishing the cut‐off levels in the used assays, with permission by the Ethics Committee of the Medical University of Vienna (EK No.: 1641/2014).

SARS‐CoV‐2 antigen/peptide‐specific antibody levels and specificities of our pediatric population were compared with those obtained in another study performed on 253 adult, SARS‐CoV‐2‐convalescent patients.^20^ SARS‐CoV‐2 infection was confirmed in all adult patients by real‐time RT‐PCR. Furthermore, patients were classified by symptom severity to 139 subjects who had a mild course of the disease and recovered at home, and 114 who experienced severe COVID‐19 and required hospitalization. The samples were collected in the same period of pandemic and in the comparable timeframe after COVID‐19 symptoms onset (pediatrics: mean 12 ± 3 weeks, 8–17 weeks; adults: mean 9 ± 2 weeks, 3–14 weeks). The same assays were used to determine the SARS‐CoV‐2 antibody response.

### 2.2 Detection of SARS‐CoV‐2‐specific antibody responses by ELISA

SARS‐CoV‐2 S protein‐ and RBD‐specific IgG was determined by ELISA as described^24^ with the following alterations. NUNC Maxisorp 96‐well plates (Thermofisher, Waltham, MA, USA) were coated with 2 µg/ml of S or RBD protein (Genscript, Leiden, Netherlands) at +4°C overnight. Plates were subsequently blocked (PBS, 2% BSA, 0.05% Tween 20) and incubated overnight with 1:50 diluted serum samples. Plates were washed and incubated for 1 hour with 1:1000 diluted HRP‐conjugated anti‐human IgG (BD, San Jose, CA, USA) and developed with ABTS (Sigma‐Aldrich, St. Louis, MO, USA). The optical density was measured at 405/492 nm with an Infinite F50 ELISA reader after 10 min (Tecan, Männedorf, Switzerland). Sera from historical controls were analyzed by ELISA and used to establish cut‐off levels.

### 2.3 Microarray containing SARS‐CoV‐2 proteins, control proteins, and synthetic S‐derived peptides

Peptides with a length of 25–30 amino acids with an overlap of approximately 5 amino acids spanning the sequence of the complete spike protein (S1 and S2) were synthesized by solid‐phase synthesis as described (Table S1).^24^

The S protein‐derived peptides as well as the following proteins (Insect cell‐expressed folded S protein, Genscript, Piscataway, NJ; HEK cell‐expressed, folded S1, Genscript; *E*. *coli*‐expressed, unfolded S1^20^; HEK cell‐expressed, folded S2, Native Antigen Company, Oxford, UK; *E*. *coli*‐expressed, unfolded S2^20^; HEK cell‐expressed, folded RBD, Genscript; *E*. *coli*‐expressed, unfolded RBD^20^) were spotted onto pre‐activated glass slides as described.^20^, ^27^, ^28^ The analysis of specific IgG levels was performed as described^20^ on sera from six representative children exhibiting different VNT titers (P16, P17, P19, P21, P23, and P26). Additionally, sera from two control children (C5 and C12) were analyzed. Pre‐pandemic sera was used for establishing cut‐off values.

### 2.4 SARS‐CoV‐2 virus neutralization test and inhibition of the RBD‐ACE2 interaction

The SARS‐CoV‐2 neutralization test (VNT) was performed as described.^19^, ^26^ To determine the inhibition of RBD to bind the ACE2 receptor by antibodies present in patients’ sera, a molecular interaction assay was used.^24^ In this assay, ELISA‐plate‐bound ACE2 was exposed to recombinant His‐tagged RBD which had been pre‐incubated with patients’ sera. Bound RBD was detected with a mouse anti‐His antibody followed by the detection with a secondary HRP‐labelled antibody. Mean optical density (O.D.) values corresponding to the amount of bound RBD were measured at 405/492 nm with an Infinite F50 ELISA reader after 10 minutes (Tecan, Männedorf, Switzerland). The ACE2 receptor served as a positive control, and an unrelated protein was used as a negative control in the blocking experiments. In each measurement, the buffer control (overlay without RBD) was subtracted as background. The measurements were done in duplicates and results are displayed as mean values with a variation of <5%. The percentages of inhibition were calculated as follows:

$$\text{Percentage inhibition}\;\left(\%\right)\;=\;\left(\text{ODnegcontrol}\;‐\;\text{ODserum}\right)/\left(\text{ODnegcontrol}‐\text{ODACE2}\right)\times 100.$$

### 2.5 Statistical analysis

All statistical analyses were performed using GraphPad Prism Version 5.00 (La Jolla, CA, USA).

Differences in immunoglobulin reactivity to proteins in different groups were determined using a two‐tailed Mann‐Whitney *U*‐test. Kruskal‐Wallis test with Dunn's post‐test was used to compare data between children, mild and severe adults. Correlations of immunoglobulin reactivity, virus neutralization titers, and percentages of blocking RBD‐ACE2 interaction were assessed by Spearman´s rank correlation coefficient. *P* values of <0.05 were considered as significant.

## 3 RESULTS

### 3.1 Characterization of studied children

Serum samples from 26 seropositive children (median age 13 years, 50% males) and 26 age‐ and gender‐matched controls were obtained from the cross‐sectional study on schoolchildren from May to July 2020.^19^ Fourteen (53.9%) seropositive children self‐reported mild acute symptoms in questionnaire whereas twelve (46.1%) did not report any symptoms in the weeks prior to the study (Table 1). One third of the control group subjects experienced and self‐reported some types of acute symptoms as well (Table 1). The vast majority of seropositive subjects (ie, 13 out of 14) experienced symptoms 5–16 weeks prior to the collection of serum. For asymptomatic patients, we assume that the infection occurred earliest at the end of February when the first SARS‐CoV‐2 cases were registered in Austria, ie, 16–20 weeks prior to the collection of serum.

**TABLE 1:** Demographic characteristics of the pediatric study population

	SARS‐CoV−2 seropositive (%)	Control group (%)
Overall	26 (100)	26 (100)
Gender (male)	13 (50)	13 (50)
Age (years)		
7–10	3 (11.6)	3 (11.6)
11–14	18 (69.2)	18 (69.2)
15–17	5 (19.2)	5 (19.2)
Self‐reported symptoms		
Overall	14 (53.9)	8 (30.8)
Period between self‐reported symptoms and study		
1–4 weeks	0	4 (15.4)
5–8 weeks	3 (11.6)	2 (7.7)
9–12 weeks	4 (15.4)	0
13–16 weeks	6 (23.0)	1 (3.9)
17–20 weeks	1 (3.9)	1 (3.9)

### 3.2 IgG levels specific for SARS‐CoV‐2 antigens are comparable regarding gender and symptoms

**FIGURE 1:**
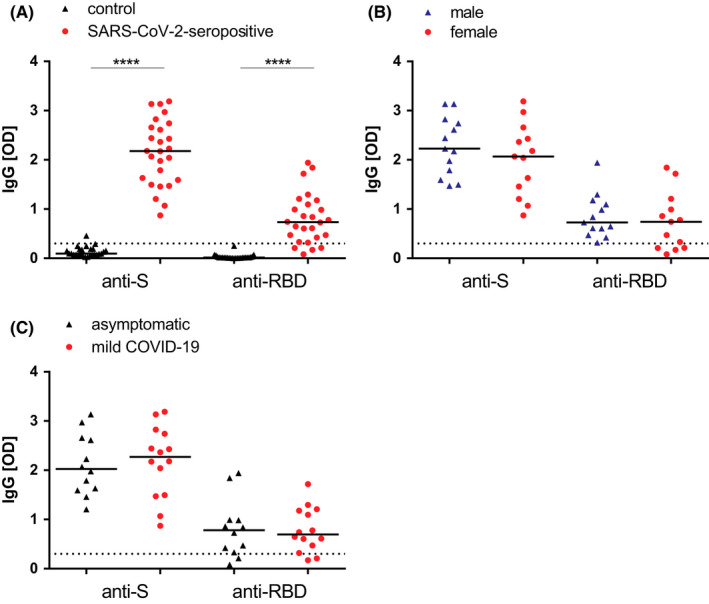
SARS‐CoV‐2‐specific IgG immune response. Anti‐S and RBD‐specific IgG levels in (A) seropositive children and control group; (B) in female versus male; and (C) asymptomatic versus mildly affected seropositive children. OD values are indicated on y‐axes. The horizontal dashed lines indicate the cut‐off levels for a positive result. Median values are indicated as horizontal bars. Significant differences are indicated (*P* value: ****<.0001)

Anti‐S and RBD‐specific IgG antibodies in sera from SARS‐CoV‐2‐seropositive children and in an age‐ and gender‐matched control group were determined (Figure 1A, Table S2). S‐specific IgG antibodies were detected in each of the seropositive children but not in the control group, except for one child (Figure 1A, Table S2: C11). Furthermore, 84.6% of the seropositive children showed RBD‐specific IgG antibodies above the cut‐off level (Figure 1A, Table S2). Conversely, none of the subjects in the control group had RBD‐specific IgG antibodies. Furthermore, IgG antibody levels mounted to S protein were significantly higher than those of RBD in seropositive children (*p *< 0.0001; Figure 1A).

All four seropositive children without RBD‐specific IgG antibodies (15.4%) were female (Table S2: P6, P8, P23, and P26). However, no significant differences in S‐ and RBD‐specific IgG antibody levels were observed in respect of gender (Figure 1B) or symptoms (Figure 1C) in the seropositive group of children.

### 3.3 IgG antibodies of COVID‐19 convalescent children are mainly directed to folded S and RBD but not to S‐derived sequential peptide epitopes

**FIGURE 2:**
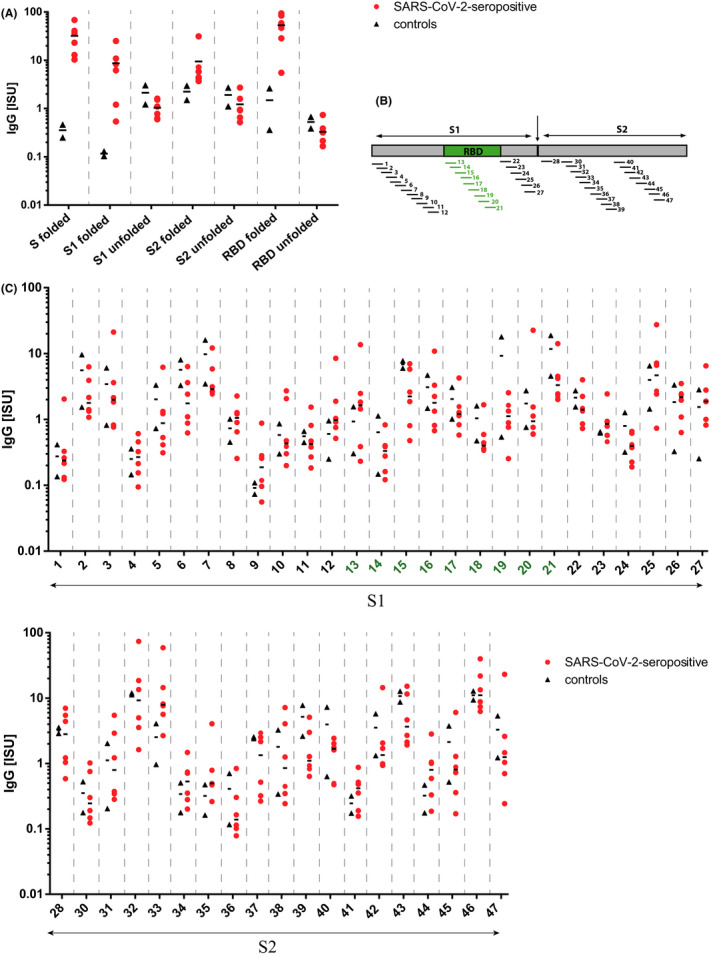
IgG reactivity of sera from representative SARS‐CoV‐2‐infected children and controls to microarrayed SARS‐CoV‐2 proteins and S‐derived peptides. Shown are IgG antibody levels (y‐axes: ISU) to SARS‐CoV‐2 proteins (x‐axis) (A) and to S‐derived peptides as indicated in the schematic overview (B) with the corresponding results (C). Median values are indicated by horizontal bars

The IgG response to a panel of microarrayed, folded, and unfolded SARS‐CoV‐2 proteins and forty‐six 25 to 30mer peptides spanning the S protein (Table S1) was assessed in a subset of six representative seropositive children exhibiting different VNT levels and two control children. Each of the tested subjects had antibodies directed to eukaryotic‐expressed, folded S (100%), and the majority showed IgG reactivity to folded RBD (ie, 5 of 6, 83.3%). Folded subdomain S1 was recognized by all subjects, whereas folded S2 was recognized by only one (17%) of the COVID‐19 convalescent children (Figure 2A). IgG antibodies to prokaryotic‐expressed, unfolded S1, S2, and RBD were not detected (Figure 2A).

IgG response to S‐derived linear peptides were in general much lower than to folded S and RBD and occurred only in certain serum samples (Figure 2B,C). Notably, the nine peptides spanning RBD were poorly recognized. Overall, an increased IgG response was only detected to peptides 32, 33, and 46 in S2 domain of the spike protein (Figure 2C) and was the most prominent for the patient with the highest VNT titer.

### 3.4 Sera of COVID‐19 convalescent children show virus‐neutralizing capacity and inhibit the RBD‐ACE2 interaction

**FIGURE 3:**
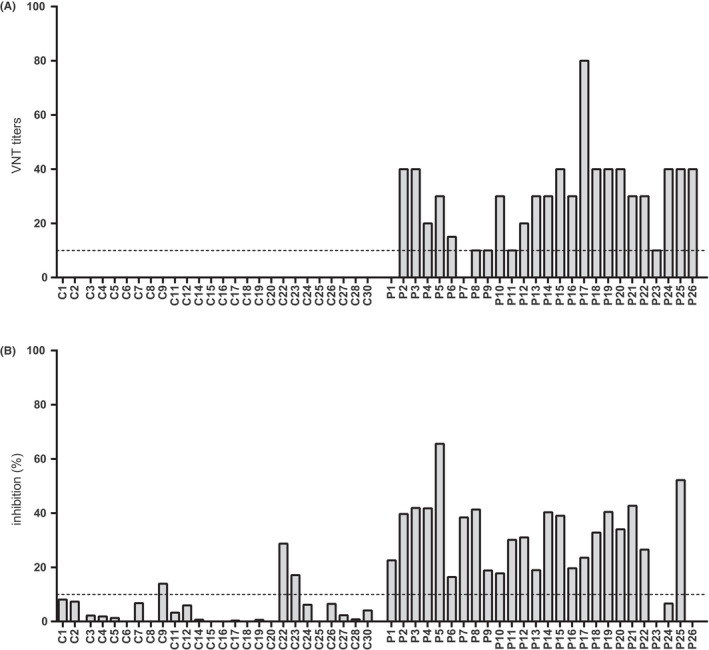
Functional capacity of the antibodies. Virus neutralization titers (A, y‐axis) and percentages of inhibition of RBD binding to ACE2 (B, y‐axis). SARS‐CoV‐2 seropositive subjects are indicated with P1‐P26 and controls with C1‐C30 (x‐axis). The experiments are done in duplicates and presented as a mean value for each subject

**FIGURE 4:**
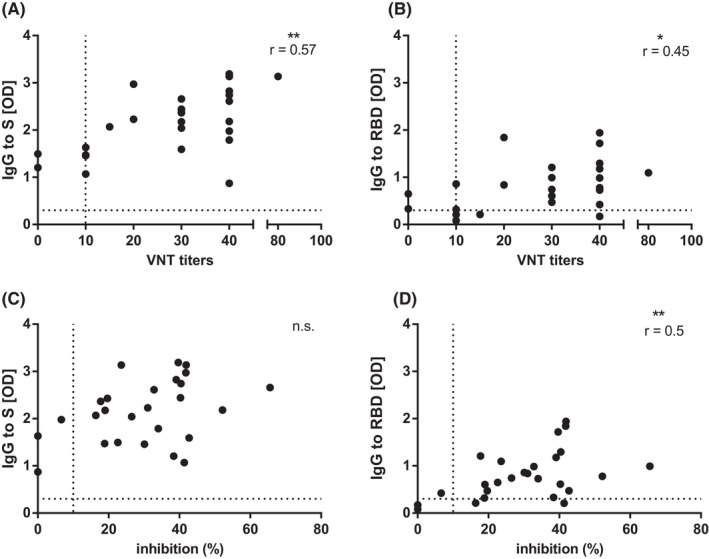
Correlation of S‐ and RBD‐specific IgG responses, virus neutralization titers, and capacity of sera to block virus receptor interaction in SARS‐CoV‐2 seropositive children (P1‐P26). Correlations of S‐ (A, C) and RBD‐specific IgG levels (B, D) (x‐axes: optical density OD levels) with VNT titers and percentages of inhibition of RBD‐binding to ACE2, respectively. Dashed lines indicate cut‐off levels. R and *P* values (*<.05, **<.001) are indicated

The functionality of the SARS‐CoV‐2‐specific antibodies in the children was investigated. The virus‐neutralization tests showed that 24 out of 26 seropositive children (92.3%) had virus‐neutralizing antibodies (Figure 3A, Table S2). Notably, the virus neutralization titers were irrespective of gender and symptom presentation (data not shown) and low. Two (7.7%) seropositive children did not show virus‐neutralizing capacity, seven (26.9%) had a VNT titer of 10–20, and sixteen (61.6%) had a titer of 30–40 (Figure 4A). Only one child (3.8%) had a virus‐neutralization titer of ≥80. All children in the control group were negative.

Blocking of RBD‐binding to ACE2 was detected in all but three seropositive children (88.5%) (Figure 3B, Table S2). Overall, sera from seropositive children inhibited RBD binding to ACE2 up to 65% with a median inhibition of 31.9% (Figure 3B). Three children (ie, C9, C22, and C23) in the control group showed more than 10% blocking of RBD binding to ACE2 (Figure 3B, Table S2).

Discordant results were found in 5 out of 26 seropositive children when comparing VNT test and assay of molecular inhibition of RBD‐ACE2 binding (Figure 3A,B). Three children of the control group who were capable to inhibit molecular interaction of RBD with ACE2 were VNT negative. Overall, the outcomes of VNT and RBD to ACE2‐binding inhibition assay were comparable in 84.6% of cases.

### 3.5 Correlation of functional assays with S‐ and RBD‐specific IgG levels in children

A correlation of antibody levels to VNT and RBD to ACE2‐binding inhibition assay was assessed in SARS‐CoV‐2 seropositive children. VNT titers showed a significant positive correlation with S‐specific IgG and to a lower extent with RBD‐specific IgG (Figure 4A,B). In contrast, the ability of antibodies to inhibit the RBD‐ACE2 interaction correlated significantly with RBD‐specific IgG but not with S‐specific IgG (Figure 4C,D). Furthermore, in the children, VNT titers and the extent of ACE2‐RBD inhibition did not correlate (data not shown).

### 3.6 Antibody responses to SARS‐CoV‐2 are comparable in pediatric and adult patients

Our population of children consisting of asymptomatic and mildly symptomatic subjects showed comparable S‐specific IgG levels by ELISA as adult subjects after severe COVID‐19, while they were significantly higher than those of adults after mild COVID‐19 (Table 2). This difference was not noted in a subset of children tested by chip technology (Table 2, lower part). RBD‐specific IgG antibody levels did not differ significantly between children and adults after mild symptoms as measured by ELISA and chip technology but were approximately twice lower than that of adults after severe COVID‐19, and the difference was significant (Table 2). The median inhibition of RBD binding to ACE2 did not differ between children and adults, whereas VNT titers were significantly lower in children. (Table 2).

**TABLE 2:** Comparison of SARS‐CoV‐2 immune response in children and adults

Analysis	Parameter	Children, mild symptoms (n = 26)	Adults, mild symptoms^a^ (n = 139)	Adults, severe symptoms^a^ (n = 114)
ELISA	IgG to S [OD]	2.17 (1.04)	1.73 (1.12) **	2.11 (0.65)
ELISA	IgG to RBD [OD]	0.73 (0.6)	0.69 (1.02)	1.42 (0.7)***
Molecular inhibition assay	Inhibition (%)	31.9 (21.2)	24.2 (33.6)	23.8 (36.9)
		Children, mild symptoms (n = 26)	Adults, mild symptoms^a^ (n = 28)	Adults, severe symptoms^a^ (n = 14)
Virus neutralization test	Titres	30 (23.8)	60 (69.1)***	320 (240)***
		Children, mild symptoms (n = 6)	Adults, mild symptoms^a^ (n = 33)	Adults, severe symptoms^a^ (n = 19)
CHIP	IgG to S folded [ISU]	29.80 (24.2)	23.02 (14.1)	50.38 (13.13)
CHIP	IgG to RBD folded [ISU]	52.73 (44.64)	64.83 (23.64)	93.62 (0.01)**

Note

Results are presented as median values with interquartile range indicated in brackets.

*P* values were calculated by Kruskal‐Wallis test with Dunn's post hoc multiple comparisons test; significant outcomes for children versus mild or severe adults are indicated (**<.01; ***<.001).

^a^ Adult population described and analyzed.^24^

When comparing the IgG response to microarrayed folded S and RBD antigens in a subset of our pediatric patients, levels were similar to those of adults with mild symptoms, but lower than those of adults after severe COVID‐19 (Table 2).

## 4 DISCUSSION

As pediatric patients mainly experience asymptomatic or a mild course of COVID‐19, understanding their antibody response to SARS‐CoV‐2 is of interest to appreciate differences to adults who are often more severely affected. We investigated the SARS‐CoV‐2‐specific IgG antibody response in 26 seropositive children and 26 age‐ and gender‐matched controls obtained from a cross‐sectional study in schoolchildren during the first wave of the COVID‐19 pandemic in Vienna, Austria.^19^ Additionally, we compared the SARS‐CoV‐2‐specific antibody response in this pediatric population with an adult convalescent population obtained in the same country at the same time.^20^

After a mild course of COVID‐19, children mounted a detectable anti‐S and RBD‐specific IgG antibody response with functional capacity of virus neutralization and ACE2‐RBD binding inhibition. Although showing generally low VNT titers, children displayed comparable IgG antibody response intensity and specificity in most of the applied assays as the adult population after a mild course of the disease, in terms of prevention of ACE2‐RBD molecular interaction and SARS‐CoV‐2 antigen and epitope recognition.

In accordance with other studies including adult and pediatric subjects,^2^, ^26^, ^29^ all SARS‐CoV‐2 seropositive‐tested subjects, as defined by the criteria used for the previous study,^19^ developed S‐specific IgG antibodies. Anti‐RBD IgG antibodies were found in 84.6% of COVID‐19 convalescent children, whereas in 15.4% of children, IgG antibodies against RBD were not detected. Using the same assay, approximately 20% of adult patients were negative for RBD‐specific IgG, with females being affected more often.^20^ Whether these discrepancies confer to the previously observed sex‐related susceptibility of COVID‐19 remains open.^30^ However, we could not find any significant gender‐related differences in the SARS‐CoV‐2‐specific antibody response in our pediatric population. This might implicate that gender‐dependent differences, responsible for SARS‐CoV‐2‐specific antibody response divergences, are not yet expressed at the young age.

In a recent adult study focusing on anti‐S1‐ and anti‐N‐specific SARS‐CoV‐2 antibody responses, significant titer differences of neutralizing antibody responses were observed between asymptomatic and mild COVID‐19‐affected individuals/patients.^18^ We could not observe such a divergence in neutralizing titers in our pediatric population, but the assays used in the studies differed. The difference between before mentioned and our study might be explained by a very mild course of the disease in our pediatric group with a generally low neutralizing titer expression or the fact that age directly correlates with more accurate antibody responses assignable to disease activity.

Using microarrayed folded and unfolded SARS‐CoV‐2 antigens, we found that IgG antibodies of COVID‐19 convalescent children were directed against folded S, S1, and RBD but not to the unfolded proteins indicating that antibodies react with conformational epitopes on the SARS‐CoV‐2 spike protein. This assumption was supported by low IgG reactivity against sequential S‐derived and, in particular, against RBD‐derived peptide epitopes among children. The same results were observed for an adult COVID‐19 population from the same region whose serum samples were obtained at the same time of the pandemic.^20^

We assessed the capability of the patients’ antibodies to prevent RBD‐ACE2 molecular interaction when RBD is pre‐incubated with patients’ sera by an in‐house assay. In our seropositive study population, inhibition levels ranged from 17.7% up to 65.6% which was in concordance with previously reported results from adult patients.^24^ Although the outcome directly correlated with anti‐RBD titers in the pediatric population, adult patients after a severe disease course showed comparable inhibition capacity despite increased RBD IgG antibody levels. This may implicate that these patients due to longer virus exposure and higher virus load mount antibodies detectable by ELISA but not functional in terms of RBD‐ACE2‐binding inhibition. Three out of 26 control patients’ sera inhibited RBD‐ACE2 molecular interaction and, therefore, were considered positive. This could be explained by cross‐reactivity with seasonal human coronaviruses. Another possible explanation is an undetected SARS‐CoV‐2 infection; however, we consider it highly unlikely as all other parameters (anti‐S and ‐RBD IgG antibodies and VNT) were negative.

Although differences in antibody responses to SARS‐CoV‐2 antigens in children and adults with indications of reduced protective serological response in younger age were previously reported,^2^ we did not observe consistent differences when comparing our results from children with an adult population assessed by same assays.^20^ We observed that mildly affected children and adults expressed a similar magnitude of RBD‐specific IgG antibody levels. Likewise, there were no big differences regarding S‐specific antibody responses in children and adults. The ability to inhibit RBD binding to ACE2 did not differ significantly between children and adults. Only VNT titers in children were lower than in adults. Despite the comparable antibody titer levels in the pediatric and adult populations, virus neutralization was, in general, more pronounced in adults in this functional assay, suggesting that other factors may underlie this observation.

Even though our study is limited only to humoral aspect of immunity, it seems that SARS‐CoV‐2 pediatric patients exhibit SARS‐CoV‐2‐specific antibody responses which are quite comparable regarding antigen, epitope recognition, and the ability to inhibit the RBD‐ACE2 interaction to that of adult patients after mild COVID‐19. This result, if confirmed in larger studies, would indicate that the mainly mild or asymptomatic course of COVID‐19 in children versus the more severe manifestations in adults is rather not due to differences regarding magnitude and quality of the specific antibody responses in children versus adults. Other factors such as different ACE2‐receptor expression, differences regarding cellular and/or innate immunity may be important.^31^

## CONFLICT OF INTEREST

Rudolf Valenta has received research grants from HVD Life‐Sciences, Vienna, Austria, WORG Pharmaceuticals, Hangzhou, China, and from Viravaxx AG, Vienna, Austria. He serves as consultant for Viravaxx AG and WORG. The other authors have no conflict of interest to declare.

## AUTHOR CONTRIBUTIONS

**Sonja Kopanja:** Conceptualization (equal); Data curation (equal); Formal analysis (equal); Investigation (equal); Methodology (equal); Writing – original draft (equal); Writing – review & editing (equal). **Pia Gattinger:** Conceptualization (equal); Data curation (equal); Formal analysis (equal); Investigation (equal); Methodology (equal); Writing – original draft (equal); Writing – review & editing (equal). **Klara Schmidthaler:** Data curation (equal); Formal analysis (equal); Methodology (equal); Writing – review & editing (equal). **Justyna Sieber:** Data curation (equal); Formal analysis (equal); Methodology (equal); Writing – review & editing (equal). **Katarzyna Niepodziana:** Methodology (equal); Writing – review & editing (equal). **Thomas Schlederer:** Methodology (equal). **Lukas Weseslindtner:** Methodology (equal); Writing – review & editing (equal). **Karin Stiasny:** Methodology (equal); Writing – review & editing (equal). **Florian Götzinger:** Data curation (equal); Writing – review & editing (equal). **Winfried F. Pickl:** Data curation (equal); Writing – review & editing (equal). **Thomas Frischer:** Data curation (equal); Funding acquisition (equal); Project administration (equal); Writing – review & editing (equal). **Rudolf Valenta:** Conceptualization (equal); Data curation (equal); Formal analysis (equal); Methodology (equal); Writing – review & editing (equal). **Zsolt Szépfalusi:** Conceptualization (equal); Data curation (equal); Formal analysis (equal); Funding acquisition (equal); Investigation (equal); Methodology (equal); Project administration (equal); Writing – original draft (equal); Writing – review & editing (equal).

## Supporting information

Table S1

Table S2

## References

[1] Fialkowski A , Gernez Y , Arya P , Weinacht KG , Kinane TB , Yonker LM . Insight into the pediatric and adult dichotomy of COVID‐19: Age‐related differences in the immune response to SARS‐CoV‐2 infection. Pediatr Pulmonol. 2020;55(10):2556‐2564. 32710693 10.1002/ppul.24981

[2] Weisberg SP , Connors TJ , Zhu Y , et al. Distinct antibody responses to SARS‐CoV‐2 in children and adults across the COVID‐19 clinical spectrum. Nat Immunol. 2021;22(1):25‐31. 33154590 10.1038/s41590-020-00826-9 PMC8136619

[3] Tönshoff B , Müller B , Elling R , et al. Prevalence of SARS‐CoV‐2 infection in children and their parents in Southwest Germany. JAMA Pediatr. 2021;175(6):586‐593. 33480966 10.1001/jamapediatrics.2021.0001 PMC7823424

[4] Zhang J‐J , Dong X , Cao Y‐Y , et al. Clinical characteristics of 140 patients infected with SARS‐CoV‐2 in Wuhan, China. Allergy. 2020;75(7):1730‐1741. 32077115 10.1111/all.14238

[5] Azkur AK , Akdis M , Azkur D , et al. Immune response to SARS‐CoV‐2 and mechanisms of immunopathological changes in COVID‐19. Allergy. 2020;75(7):1564‐1581. 32396996 10.1111/all.14364 PMC7272948

[6] De Jacobis IT , Vona R , Cittadini C , et al. Clinical characteristics of children infected with SARS‐CoV‐2 in Italy. Ital J Pediatr. 2021;47(1):90. 33858472 10.1186/s13052-021-01045-0 PMC8047584

[7] Castagnoli R , Votto M , Licari A , et al. Severe acute respiratory syndrome coronavirus 2 (SARS‐CoV‐2) infection in children and adolescents: a systematic review. JAMA Pediatr. 2020;174(9):882‐889. 32320004 10.1001/jamapediatrics.2020.1467

[8] Öcal Demi̇r S , Tosun Ö , Öztürk K , et al. SARS‐CoV‐2 associated multisystem inflammatory syndrome in children (MIS‐C). A Single Center’s Experience. Minerva Pediatr. 2021. 10.23736/S2724-5276.21.06327-8 33890746

[9] Aguilera‐Alonso D , Murias S , Martínez‐de‐Azagra Garde A , et al. Prevalence of thrombotic complications in children with SARS‐CoV‐2. Arch Dis Child. 2021;106(11):1129‐1132. 33931403 10.1136/archdischild-2020-321351

[10] Carsetti R , Zaffina S , Piano Mortari E , et al. Different innate and adaptive immune responses to SARS‐CoV‐2 infection of asymptomatic, mild, and severe cases. Front Immunol. 2020;11:610300. 33391280 10.3389/fimmu.2020.610300 PMC7772470

[11] Jamilloux Y , Henry T , Belot A , et al. Should we stimulate or suppress immune responses in COVID‐19? Cytokine and anti‐cytokine interventions. Autoimmun Rev. 2020;19(7):102567. 32376392 10.1016/j.autrev.2020.102567 PMC7196557

[12] Shang J , Ye G , Shi KE , et al. Structural basis of receptor recognition by SARS‐CoV‐2. Nature. 2020;581(7807):221‐224. 32225175 10.1038/s41586-020-2179-y PMC7328981

[13] Saheb Sharif‐Askari N , Saheb Sharif‐Askari F , Alabed M , et al. Airways expression of SARS‐CoV‐2 receptor, ACE2, and TMPRSS2 is lower in children than adults and increases with smoking and COPD. Mol Ther Methods Clin Dev. 2020;18:1‐6. 32537478 10.1016/j.omtm.2020.05.013 PMC7242205

[14] Pavel AB , Wu J , Renert‐Yuval Y , et al. SARS‐CoV‐2 receptor ACE2 protein expression in serum is significantly associated with age. Allergy. 2021;76(3):875‐878. 32726474 10.1111/all.14522 PMC8278339

[15] Vuille‐Dit‐Bille RN , Liechty KW , Verrey F , Guglielmetti LC . SARS‐CoV‐2 receptor ACE2 gene expression in small intestine correlates with age. Amino Acids. 2020;52(6–7):1063‐1065. 32627059 10.1007/s00726-020-02870-z PMC7335412

[16] Li Y , Zhou W , Yang L , You R . Physiological and pathological regulation of ACE2, the SARS‐CoV‐2 receptor. Pharmacol Res. 2020;157:104833. 32302706 10.1016/j.phrs.2020.104833 PMC7194807

[17] Rostad CA , Chahroudi A , Mantus G , et al. Quantitative SARS‐CoV‐2 serology in children with multisystem inflammatory syndrome (MIS‐C). Pediatrics. 2020;146(6):e2020018242. 32879033 10.1542/peds.2020-018242

[18] Lei Q , Li Y , Hou H‐Y , et al. Antibody dynamics to SARS‐CoV‐2 in asymptomatic COVID‐19 infections. Allergy. 2021;76(2):551‐561. 33040337 10.1111/all.14622 PMC7675426

[19] Szépfalusi Z , Schmidthaler K , Sieber J , et al. Lessons from low seroprevalence of SARS‐CoV‐2 antibodies in schoolchildren: a cross‐sectional study. Pediatr Allergy Immunol. 2021;32(4):762‐770. 33512035 10.1111/pai.13459 PMC8013685

[20] Gattinger P , Niespodziana K , Stiasny K , et al. Neutralization of SARS‐CoV‐2 requires antibodies against conformational receptor‐binding domain epitopes. Allergy. 2022;77(1):230‐242. 34453317 10.1111/all.15066 PMC8653362

[21] Zost SJ , Gilchuk P , Case JB , et al. Potently neutralizing and protective human antibodies against SARS‐CoV‐2. Nature. 2020;584(7821):443‐449. 32668443 10.1038/s41586-020-2548-6 PMC7584396

[22] Baum A , Fulton BO , Wloga E , et al. Antibody cocktail to SARS‐CoV‐2 spike protein prevents rapid mutational escape seen with individual antibodies. Science. 2020;369(6506):1014‐1018. 32540904 10.1126/science.abd0831 PMC7299283

[23] Zhang Y , Yang Z , Tian S , et al. A newly identified linear epitope on non‐RBD region of SARS‐CoV‐2 spike protein improves the serological detection rate of COVID‐19 patients. BMC Microbiol. 2021;21(1):194. 34174835 10.1186/s12866-021-02241-y PMC8234764

[24] Gattinger P , Borochova K , Dorofeeva Y , et al. Antibodies in serum of convalescent patients following mild COVID‐19 do not always prevent virus‐receptor binding. Allergy. 2021;76(3):878‐883. 32734595 10.1111/all.14523 PMC7984338

[25] Kratzer B , Trapin D , Ettel P , et al. Immunological imprint of COVID‐19 on human peripheral blood leukocyte populations. Allergy. 2021;76(3):751‐765. 33128792 10.1111/all.14647 PMC7984452

[26] Koblischke M , Traugott MT , Medits I , et al. Dynamics of CD4 T cell and antibody responses in COVID‐19 patients with different disease severity. Front Med. 2020;7:592629. 10.3389/fmed.2020.592629 PMC7686651 33262993

[27] Niespodziana K , Stenberg‐Hammar K , Megremis S , et al. PreDicta chip‐based high resolution diagnosis of rhinovirus‐induced wheeze. Nat Commun. 2018;9(1):2382. 29915220 10.1038/s41467-018-04591-0 PMC6006174

[28] Gallerano D , Wollmann E , Lupinek C , et al. HIV microarray for the mapping and characterization of HIV‐specific antibody responses. Lab Chip. 2015;15(6):1574‐1589. 25648429 10.1039/c4lc01510j

[29] Wang Y , Zhang LU , Sang L , et al. Kinetics of viral load and antibody response in relation to COVID‐19 severity. J Clin Invest. 2020;130(10):5235‐5244. 32634129 10.1172/JCI138759 PMC7524490

[30] Takahashi T , Ellingson MK , Wong P , et al. Sex differences in immune responses that underlie COVID‐19 disease outcomes. Nature. 2020;588(7837):315‐320. 32846427 10.1038/s41586-020-2700-3 PMC7725931

[31] Cristiani L , Mancino E , Matera L , et al. Will children reveal their secret? The coronavirus dilemma. Eur Respir J. 2020;55(4):2000749. 32241833 10.1183/13993003.00749-2020 PMC7113798

