# Supplementary material for: Characterization of the antibody response to SARS‐CoV‐2 in a mildly affected pediatric population
Source: Pediatr Allergy Immunol. 2022 Feb 21;33(2):e13737. doi: 10.1111/pai.13737 (PMC9115525; doi:10.1111/pai.13737)
Supplement: Supplementary file 1 — Table S1 [file PAI-33-0-s001.docx]

Supplementary Table 1. SARS-CoV-2 derived proteins and spike protein-derived peptides

| **Protein** | **Expression system** | **Source** | **Molecular weight [kDa]** |
| --- | --- | --- | --- |
| S (folded) | Insect cells | Genscript | 135 |
| S1 (folded) | HEK cells | Genscript | 79 |
| S1 (folded) | Insect cells | Genscript | 79 |
| S1 (unfolded) | *E.coli* | in-house expression | 76 |
| S2 (folded) | HEK cells | Native Antigen Company | 60 |
| S2 (unfolded) | *E.coli* | in-house expression | 56 |
| RBD (folded) | HEK cells | Genscript | 30 |
| RBD (unfolded) | *E. coli* | in-house expression | 62 |
| NP (folded) | HEK cells | Biovendor | 46 |
| NP (unfolded) | *E.coli* | Biovendor | 46 |
| **Peptide ID** | **Amino acid sequence** | **No. of amino acids** | **Molecular weight [Da]** |
| **1** | PLVSSQCVNLTTRTQLPPAYTNSFTRGVYY | 30 | 3377.8 |
| **2** | RGVYYPDKVFRSSVLHSTQDLFLPFFSNVT | 30 | 3520.9 |
| **3** | FSNVTWFHAIHVSGTNGTKRFDNPVLPFND | 30 | 3418.7 |
| **4** | LPFNDGVYFASTEKSNIIRGWIFGTTLDS | 29 | 3249.6 |
| **5** | TLDSKTQSLLIVNNATNVVIKVCEFQFCND | 30 | 3357.8 |
| **6** | QFCNDPFLGVYYHKNNKSWMESEFRVYSSA | 30 | 3648.0 |
| **7** | VYSSANNCTFEYVSQPFLMDLEGKQGNFKN | 30 | 3431.8 |
| **8** | GNFKNLREFVFKNIDGYFKIYSKHTPINLV | 30 | 3603.1 |
| **9** | PINLVRDLPQGFSALEPLVDLPIGINITR | 29 | 3171.7 |
| **10** | NITRFQTLLALHRSYLTPGDSSSGWTAGAA | 30 | 3192.5 |
| **11** | TAGAAAYYVGYLQPRTFLLKYNENGTITDA | 30 | 3282.6 |
| **12** | TITDAVDCALDPLSETKCTLKSFTVEKGIY | 30 | 3262.7 |
| **13** | EKGIYQTSNFRVQPTESIVRFPNITNLC | 28 | 3255.7 |
| **14** | FNATRFASVYAWNRKRISNCVADYS | 25 | 2940.2 |
| **15** | VADYSVLYNSASFSTFKCYGVSPTK | 25 | 2735.0 |
| **16** | VSPTKLNDLCFTNVYADSFVIRGDEVRQIA | 30 | 3371.8 |
| **17** | VRQIAPGQTGKIADYNYKLPDDFTGCVIAW | 30 | 3340.8 |
| **18** | CVIAWNSNNLDSKVGGNYNYLYRLFRKSNL | 30 | 3522.9 |
| **19** | KPFERDISTEIYQAGSTPCNGVEGF | 25 | 2746.0 |
| **20** | GVEGFNCYFPLQSYGFQPTNGVGYQPYRVV | 30 | 3387.7 |
| **21** | PYRVVVLSFELLHAPATVCGPKKSTNLVKN | 30 | 3281.9 |
| **22** | NLVKNKCVNFNFNGLTGTGVLTESNKKFL | 29 | 3200.7 |
| **23** | PFQQFGRDIADTTDAVRDPQTLEILDIT | 28 | 3176.4 |
| **24** | ILDITPCSFGGVSVITPGTNTSNQVAVLY | 29 | 2967.3 |
| **25** | AVLYQDVNCTEVPVAIHADQLTPTWRVYST | 30 | 3390.8 |
| **26** | RVYSTGSNVFQTRAGCLIGAEHVNNSYECD | 30 | 3291.5 |
| **27** | SYECDIPIGAGICASYQTQTNSP*RRAR*SVA | 30 | 3215.5 |
| **28** | TMSLGAENSVAYSNNSIANNSIAIPTNFTI | 30 | 3115.4 |
| **30** | TSVDCTMYICGDSTECSNLLLQYGSFCTQL | 30 | 3296.7 |
| **31** | FCTQLNRALTGIAVEQDKNTQEVFAQVKQI | 30 | 3393.8 |
| **32** | QVKQIYKTPPIKDFGGFNFSQILPDPSK | 28 | 3193.6 |
| **33** | PDPSKPSKRSFIEDLLFNKVTLADAGFIKQ | 30 | 3362.8 |
| **34** | GFIKQYGDCLGDIAARDLICAQKFNGLTVL | 30 | 3243.7 |
| **35** | TDEMIAQYTSALLAGTITSGW | 21 | 2229.4 |
| **36** | ITSGWTFGAGAALQIPFAMQMAYRFNGIGV | 30 | 3176.7 |
| **37** | NGIGVTQNVLYENQKLIANQFNSAIGKIQD | 30 | 3290.6 |
| **38** | GKIQDSLSSTASALGKLQDVVNQNAQALNT | 30 | 3072.3 |
| **39** | LNTLVKQLSSNFGAISSVLNDILSRLDK | 28 | 3046.5 |
| **40** | LDKVEAEVQIDRLITGRLQSLQTYVTQQ | 28 | 3245.6 |
| **41** | YVTQQLIRAAEIRASANLAATKMSECVL | 28 | 3051.5 |
| **42** | CVLGQSKRVDFCGKGYHLMSFPQSAPH | 27 | 2993.4 |
| **43** | PHGVVFLHVTYVPAQEKNFTTAPAICHDGK | 30 | 3277.7 |
| **44** | CHDGKAHFPREGVFVSNGTHWFVTQRNFYE | 30 | 3566.9 |
| **45** | RNFYEPQIITTDNTFVSGNC | 20 | 2319.5 |
| **46** | NNTVYDPLQPELDSFKEELDKYFKNHT | 27 | 3285.5 |
| **47** | FKNHTSPDVDLGDISGINASVVNIQKEI | 28 | 3011.3 |
