# Supplementary material for: Characterization of the antibody response to SARS‐CoV‐2 in a mildly affected pediatric population
Source: Pediatr Allergy Immunol. 2022 Feb 21;33(2):e13737. doi: 10.1111/pai.13737 (PMC9115525; doi:10.1111/pai.13737)
Supplement: Supplementary file 2 — Table S2 [file PAI-33-0-s002.docx]

Supplementary Table 2. Serological characterization of pediatric population and historic control sera from adults.

| **Patient ID^1^** | **age**  **(years)** | **gender^2^** | **symptoms^3^** | **IgG to S**  **(OD)^4^** | **IgG to RBD**  **(OD) ^5^** | **inhibition**  **(%)^6^** | **WANTAI (OD)^7^** | **VNT (titer)^8^** |
| --- | --- | --- | --- | --- | --- | --- | --- | --- |
| **P1** | 7 | M | yes | 1.50 | 0.65 | 22.60 | 0.69 | <10 |
| **P2** | 13 | F | yes | 3.19 | 1.72 | 39.70 | 3.75 | 40 |
| **P3** | 10 | M | no | 3.14 | 1.94 | 41.90 | 3.96 | 40 |
| **P4** | 17 | F | no | 2.97 | 1.84 | 41.80 | 4.06 | 20 |
| **P5** | 13 | F | no | 2.66 | 0.99 | 65.60 | 3.97 | 30 |
| **P6** | 13 | F | no | 2.07 | 0.21 | 16.40 | 3.42 | 15 |
| **P7** | 13 | F | no | 1.21 | 0.33 | 38.40 | 1.48 | <10 |
| **P8** | 13 | F | yes | 1.07 | 0.21 | 41.30 | 2.07 | 10 |
| **P9** | 13 | M | yes | 1.47 | 0.32 | 18.90 | 3.90 | 10 |
| **P10** | 13 | F | yes | 2.36 | 1.21 | 17.70 | 3.65 | 30 |
| **P11** | 12 | F | no | 1.46 | 0.86 | 30.10 | 3.67 | 10 |
| **P12** | 13 | M | no | 2.23 | 0.84 | 31.00 | 3.99 | 20 |
| **P13** | 14 | M | yes | 2.18 | 0.61 | 19.00 | 3.62 | 30 |
| **P14** | 13 | M | yes | 2.44 | 0.61 | 40.30 | 3.19 | 30 |
| **P15** | 12 | M | yes | 2.83 | 1.18 | 39.10 | 3.40 | 40 |
| **P16** | 16 | F | yes | 2.43 | 0.47 | 19.70 | 3.55 | 30 |
| **P17** | 13 | M | yes | 3.14 | 1.09 | 23.60 | 3.58 | ≥80 |
| **P18** | 7 | M | no | 2.61 | 0.99 | 32.80 | 3.53 | 40 |
| **P19** | 17 | M | yes | 2.74 | 1.30 | 40.40 | 3.82 | 40 |
| **P20** | 11 | M | no | 1.79 | 0.73 | 34.00 | 3.82 | 40 |
| **P21** | 13 | M | no | 1.59 | 0.47 | 42.70 | 3.88 | 30 |
| **P22** | 13 | F | yes | 2.04 | 0.74 | 26.50 | 3.63 | 30 |
| **P23** | 13 | F | no | 1.63 | 0.08 | -0.40 | 0.27 | 10 |
| **P24** | 11 | M | no | 1.98 | 0.42 | 6.70 | 3.90 | 40 |
| **P25** | 16 | F | yes | 2.18 | 0.78 | 52.20 | 3.94 | 40 |
| **P26** | 15 | F | yes | 0.87 | 0.17 | -6.20 | 0.99 | 40 |
| **C1** | 12 | F | yes | 0.10 | 0.02 | 8.10 | -0.02 | <10 |
| **C2** | 13 | F | no | 0.09 | 0.01 | 7.30 | 0.07 | <10 |
| **C3** | 7 | M | yes | 0.06 | 0.01 | 2.20 | 0.01 | <10 |
| **C4** | 10 | M | no | 0.13 | 0.01 | 1.90 | -0.04 | <10 |
| **C5** | 17 | F | no | 0.05 | 0.01 | 1.30 | 0.03 | <10 |
| **C6** | 13 | F | no | 0.29 | 0.00 | -6.30 | 0.09 | <10 |
| **C7** | 13 | F | no | 0.16 | 0.02 | 6.80 | 0.00 | <10 |
| **C8** | 13 | F | no | 0.19 | 0.03 | -1.80 | 0.05 | <10 |
| **C9** | 13 | F | no | 0.07 | 0.03 | 14.00 | -0.15 | <10 |
| **C11** | 13 | M | no | 0.46 | 0.25 | 3.30 | 0.04 | <10 |
| **C12** | 13 | F | no | 0.04 | 0.01 | 5.90 | 0.01 | <10 |
| **C14** | 16 | F | yes | 0.19 | 0.03 | 0.70 | 0.01 | <10 |
| **C15** | 14 | M | no | 0.06 | 0.02 | -1.30 | 0.08 | <10 |
| **C16** | 12 | M | no | 0.06 | 0.01 | -11.40 | 0.01 | <10 |
| **C17** | 17 | M | no | 0.07 | 0.01 | 0.40 | -0.03 | <10 |
| **C18** | 13 | F | no | 0.19 | 0.02 | -9.80 | -0.04 | <10 |
| **C19** | 13 | M | no | 0.15 | 0.02 | 0.60 | -0.03 | <10 |
| **C20** | 11 | M | no | 0.18 | 0.02 | -0.90 | 0.01 | <10 |
| **C22** | 7 | M | yes | 0.25 | 0.02 | 28.70 | 0.02 | <10 |
| **C23** | 11 | M | yes | 0.07 | 0.02 | 17.10 | 0.06 | <10 |
| **C24** | 13 | F | no | 0.25 | 0.01 | 6.20 | -0.08 | <10 |
| **C25** | 16 | F | yes | 0.03 | 0.02 | -10.60 | 0.09 | <10 |
| **C26** | 15 | F | yes | 0.02 | 0.01 | 6.50 | -0.03 | <10 |
| **C27** | 13 | M | no | 0.10 | 0.07 | 2.30 | -0.03 | <10 |
| **C28** | 13 | M | no | 0.10 | 0.06 | 0.80 | 0.00 | <10 |
| **C30** | 13 | M | yes | 0.07 | 0.07 | 4.10 | -0.01 | <10 |
|  |  |  |  |  |  |  |  |  |
| **HC1** | 18 | M | n.a | 0.03 | 0.01 | -4.60 | n.d. | n.d. |
| **HC2** | 36 | M | n.a | 0.02 | 0.02 | -5.70 | n.d. | n.d. |
| **HC3** | 29 | M | n.a | 0.05 | 0.02 | -4.80 | n.d. | n.d. |
| **HC4** | 38 | M | n.a | 0.07 | 0.01 | 0.30 | n.d. | n.d. |
| **HC5** | 28 | F | n.a | 0.07 | 0.01 | 9.30 | n.d. | n.d. |
| **HC6** | 28 | F | n.a | 0.06 | 0.01 | 0.30 | n.d. | n.d. |
| **HC7** | 39 | F | n.a | 0.25 | 0.05 | 1.10 | n.d. | n.d. |
| **HC8** | 56 | M | n.a | 0.69 | 0.05 | 4.10 | n.d. | n.d. |
| **HC9** | 69 | M | n.a | 0.07 | 0.01 | -3.80 | n.d. | n.d. |

1 P1-P26= seropositive patients, C1-C26= seronegative controls as defined by the criteria used at initial screening (Szépfalusi et al, PAI 2020); HC1-HC9= historic controls

2 F= female, M = male

3 self-reported symptoms

4 IgG reactivity to S determined by ELISA, cut-off value = 0.3

5 IgG reactivity to RBD determined by ELISA, cut-off value = 0.3

6 Inhibition of binding of RBD to ACE2, cut-off value = 10%

7 Wantai SARS-CoV-2 total Ab ELISA (Beijing Wantai Biological Pharmacy Enterprise), cut-off value = 0.18

8 Virus neutralization test, cut-off value = 10

Abbr.: n.d. not done, n.a. not applicable, O.D. optical density
